# Multivitamin/Multimineral Supplementation Prevents or Reverses Decline in Vitamin Biomarkers and Cellular Energy Metabolism in Healthy Older Men: A Randomized, Double-Blind, Placebo-Controlled Study

**DOI:** 10.3390/nu15122691

**Published:** 2023-06-09

**Authors:** Alexander J. Michels, Judy A. Butler, Sandra L. Uesugi, Ken Lee, Balz B. Frei, Gerd Bobe, Kathy R. Magnusson, Tory M. Hagen

**Affiliations:** 1 Linus Pauling Institute, Oregon State University, Corvallis, OR 97331, USA; alexander.michels@oregonstate.edu (A.J.M.); judy.a.butler@oregonstate.edu (J.A.B.); sandra.uesugi@oregonstate.edu (S.L.U.); leekenn@oregonstate.edu (K.L.); balz.frei@oregonstate.edu (B.B.F.); gerd.bobe@oregonstate.edu (G.B.); kathy.magnusson@oregonstate.edu (K.R.M.); 2 Department of Biochemistry and Biophysics, College of Science, Oregon State University, Corvallis, OR 97331, USA; 3 Department of Animal and Rangeland Sciences, College of Agriculture, Oregon State University, Corvallis, OR 97331, USA; 4 Department of Biomedical Sciences, Carlson College of Veterinary Medicine, Oregon State University, Corvallis, OR 97331, USA

**Keywords:** vitamin, mineral, dietary supplements, multivitamin, micronutrient status, healthy aging, O_2_ consumption

## Abstract

Despite the reported prevalence of micronutrient deficiencies in older adults, it is not yet established whether multivitamin/multimineral (MV/MM) supplements improve blood micronutrient status in individuals over the age of 65. Therefore, a cohort of 35 healthy men (>67 years) was recruited for an MV/MM supplementation trial. The primary endpoint was, as an indicator of micronutrient status, changes in blood micronutrient biomarkers from baseline to at least six months of supplementation with MV/MM or placebo. The secondary endpoint was basal O_2_ consumption in monocytes as an indicator of cellular metabolism. MV/MM supplementation improved blood concentrations of pyridoxal phosphate, calcifediol, α-tocopherol, and β-carotene concentrations throughout the cohort. By contrast, those in the placebo group generally showed declines in blood vitamin concentrations and an increased prevalence of suboptimal vitamin status during the study period. On the other hand, MV/MM supplementation did not significantly affect blood mineral concentrations, i.e., calcium, copper, iron, magnesium, and zinc. Interestingly, MV/MM supplementation prevented the decline in monocyte O_2_ consumption rate. Overall, MV/MM use improves or prevents declines in vitamin, but not mineral, status and limits declines in cellular O_2_ consumption, which may have important implications for metabolism and immune health in healthy older men.

## 1. Introduction

A balanced diet that is high in fruits and vegetables provides many of the micronutrients that humans need for normal metabolism and physiological function. However, even in the United States and other economically advantaged countries, large nutritional survey data indicate that a healthy diet may not adequately provide sufficient quantities of certain vitamins and minerals [1,2]. Older adults, especially those ≥65 years of age, are at increased risk for these micronutrient inadequacies [3,4,5]. Longitudinal surveys suggest that as a person ages, macronutrient consumption, as reported by caloric intake, declines [6], which indicates fewer micronutrients consumed as well. This is evidenced by the relatively high proportion of individuals aged 61 years and older not meeting the Estimated Average Requirement (EAR) for several vitamins and minerals, as set by the Food and Nutrition Board of the National Academies of Sciences Engineering and Medicine [7].

Poor micronutrient status, which is determined by vitamin and mineral concentrations in the blood, is also more likely with advanced age [8]. It is presumed that this is a result of changes in dietary habits, physiological changes, or a combination of these factors. For example, both food choices and changes in nutrient absorption may decline as a result of poor oral health, gastrointestinal pH changes, chronic low-grade inflammation of the gut (i.e., atrophic gastritis), co-morbid diseases, polypharmacy, or loss of taste and smell, and any combination of these factors [9,10,11,12,13,14,15,16]. There is also the possibility that advancing age may lead to heightened micronutrient utilization, making it more difficult for older adults to maintain blood nutrient levels. Acute or chronic inflammatory events, which often plague older individuals, may reduce the circulating concentrations of several vitamins and minerals [17,18,19,20].

Thus, it is commonly reported that food alone is often insufficient to sustain the metabolic requirements of older adults [5,21,22,23]. This provides a rationale for recommending the use of a daily multivitamin/multimineral (MV/MM) supplement, not only to meet the micronutrient needs of older individuals but to provide them in quantities and forms that are more bioavailable [23]. However, there is only limited evidence that MV/MM supplements improve the micronutrient status of adults above the age of 50 [24,25], and scarce reports for individuals above the age of 65. Moreover, much of the evidence gathered on MV/MM supplements to date has focused on a wide range of ages and ignored gender differences. Furthermore, in those studies that have included older adults, there were no attempts to exclude participants with potentially confounding age-associated co-morbidities (e.g., cardiovascular diseases, diabetes, metabolic syndrome, gastrointestinal malabsorption diseases, smoking, and/or cancer). Finally, many studies on MV/MM supplements focus on determining the reduction in disease risk and not the more fundamental question of improving micronutrient status [26,27,28,29,30,31].

Based on the aforementioned confounding factors, we conducted a double-blind, randomized, placebo-controlled trial in a cohort of healthy older men (averaging 70 years of age at the start of the trial). These participants were purposely selected with few age-associated pathophysiologies. These strict inclusion criteria were designed to eliminate as many variables as possible so that age-associated changes to micronutrient status may be discerned. Men were chosen for this study because diet and lifestyle behaviors are more likely to adversely impact micronutrient status in this sex with age [32]. The primary objective of this study was to determine whether a daily MV/MM supplement taken for 6 months could improve blood micronutrient values in these individuals. As a secondary outcome, mitochondrial respiratory function in peripheral blood mononuclear cells (PBMCs) was measured to determine if nutrition status influenced metabolic energy production. Herein, the data show that healthy older adult men responded positively to multivitamin supplementation, despite having few underlying vitamin deficiencies before the supplementation began. Additionally, these changes to micronutrient status may have a connection to the improved metabolic function observed in those taking the MV/MM supplement.

## 2. Materials and Methods

### 2.1. Study Design

This was a single-center, two-armed, parallel, randomized, double-blind study designed to determine the effect of daily oral intake of an MV/MM supplement (i.e., Centrum Silver™ Men’s Formula) on vitamin and mineral status in healthy male older adults after 6 months of supplementation. This study was conducted in accordance with the guidelines set by the Declaration of Helsinki, and the study design was approved by the Institutional Review Board at Oregon State University (Study #7158). The study is registered at clinicaltrials.gov (#NCT03004807). A brief description of the study follows, with details provided in Supplementary Methods. Full protocol available upon request.

### 2.2. Participants

To be eligible, participants needed to be generally healthy, above the age of 67, and willing to refrain from taking any dietary supplements containing any of the nutrients provided by the MV/MM supplement itself (Table S1). An exception was made for vitamin D because it is commonly prescribed or recommended by physicians in the Pacific Northwest to treat vitamin D deficiency. Participants who were taking MV/MM supplements before enrollment were required to wait at least 2 months without using any supplements before engaging in the study protocol. Additional exclusion criteria are listed in Supplementary Methods.

**Figure 1:**
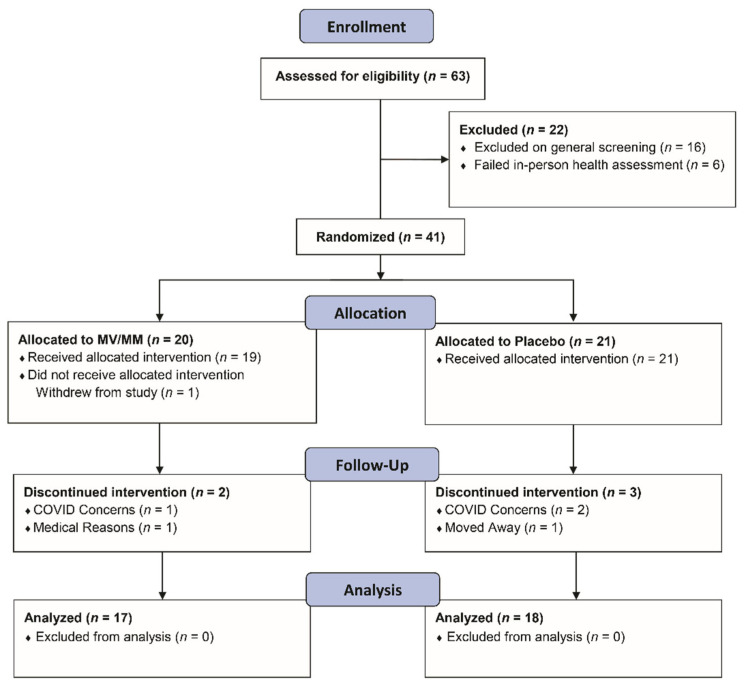
Flow diagram showing the progression of study participants—healthy men over the age of 67—through the study protocol.

Participants were recruited from Corvallis, Oregon, and surrounding communities via email, phone calls, and flyers starting in July 2018. Volunteers in Oregon State University’s Center for Healthy Aging Research LIFE registry were also contacted to determine their interest and eligibility. Respondents were assessed for eligibility until February 2020, when the target of at least 40 enrolled participants was reached (Figure 1). Eligibility assessment included an initial visit to the Clinical Research Center at the Linus Pauling Institute to obtain written informed consent and health evaluation that included anthropomorphic measures and blood chemistry (Table 1). Informed consent was obtained from all participants involved in the study.

Those that met the eligibility criteria were assigned to study treatment groups by the study statistician (Bobe) using a randomized block design, blocking for prior MV/MM use (yes/no), and baseline BMI category (normal, overweight, and obese). Prior to engaging in study activities, participants provided baseline blood samples. Once blood was collected, tablets were distributed, and other study activities were conducted as described below and in Supplementary Methods.

### 2.3. Primary and Secondary Outcomes

Participants were instructed to consume tablets provided by Pfizer-GSK Consumer Healthcare (now Haleon), which contained the MV/MM supplement or a matched placebo. These were provided in 90-count bottles that were sealed and coded without identifying marks as to their contents. Participants were advised to take one of the assigned tablets every day with or without a meal. The primary study endpoint of this trial was to assess the change in metabolic function, primarily by measuring the oxygen consumption rate in isolated PBMCs.

To measure these outcomes, participants provided fasting overnight blood samples before and at the end of the supplementation period. As described in detail in the Supplementary Methods, samples were analyzed for blood lipids (triglycerides, total cholesterol, HDL, VLDL, and LDL), a comprehensive metabolic panel (CMP), Hb-A1C, and ferritin using standard clinical assays (Student Health Services, Oregon State University, Corvallis, OR, a CLIA-certified diagnostic service laboratory). Serum concentrations of vitamins D and B_12_, plasma vitamin B_6_, and red blood cell folate (B_9_) were determined by Quest Diagnostics (Seattle, WA, USA), a CLIA-certified diagnostic laboratory. Other vitamins were measured at the Linus Pauling Institute’s Analytical Services Core Laboratory. Minerals were analyzed in the W.M. Keck Collaboratory for Plasma Spectrometry in OSU’s College of Earth, Ocean, and Atmospheric Sciences.

Plasma carotenoids (retinol, β-carotene, lycopene, lutein) and vitamins K and E (α-tocopherol) were analyzed using a Xevo MS/MS mass spectrometer (Waters Xevo TQD, Milford, MA, USA) housed in LPI’s Analytical Services Core laboratory and quantitated using authentic standards: α-tocopherol acetate and carotenoids for vitamin E and carotenoids [33,34], respectively, and d_4_-phylloquinone for vitamin K [35]. Vitamin C was measured using the LPI’s Analytical Services Core facilities, as described previously, by HPLC coupled to electrochemical detection [36]. Plasma copper, zinc, iron, magnesium, and selenium were analyzed by inductively-coupled plasma-optical-emission spectroscopy (ICP-OES) using a Spectros Arcos instrument [37].

Blood micronutrient concentrations were used to identify status categories, specifically deficiency and suboptimal status (and inadequacies in the case of calcidiol), as defined in previous reports [38,39,40,41,42,43,44,45,46,47]. These cutoff values are listed here for reference. For serum retinol (vitamin A), deficiency is <0.7 μM, and suboptimal status is <1.05 μM. For serum pyridoxal 5-phosphate (a biomarker for vitamin B_6_), deficiency is <4.9 ng/mL, and suboptimal status is <8.9 ng/mL. Red blood cell folate deficiency levels are <150 ng/mL, with suboptimal status <400 ng/mL. For serum cobalamin (i.e., vitamin B_12_), concentrations <200 pg/mL indicate a deficiency, and <500 pg/mL are suboptimal. For plasma ascorbic acid (vitamin C), deficiency is <11.4 μM, while suboptimal status is <50 μM. For calcidiol (i.e., vitamin D), <12 ng/mL is defined as a deficiency, <20 ng/mL is defined as insufficiency, and <30 ng/mL is considered suboptimal. For α-tocopherol, deficiency is <12 μM, and suboptimal is considered <30 μM. For plasma phylloquinone (vitamin K_1_), deficiency is <0.5 μM, and suboptimal status is <1.0 μM.

For studies associated with mitochondrial metabolic assessment, PBMCs were isolated from 40 mL of heparinized whole blood within 4 h of collection. High-resolution O_2_ consumption measurements of mononuclear cells were conducted using the OROBOROS Oxygraph-2k (OROBOROS Instruments, Innsbruck, Austria) and recorded by DatLab 4 software [48]. Intact PBMCs were resuspended in miR05 respiration buffer to determine the initial oxygen consumption “Total Cellular” rate. PBMCs were then permeabilized by the addition of digitonin to establish O_2_ consumption from organelles. The amount of digitonin added was determined empirically based on the negation of basal O_2_ consumption rate. Sequential addition of substrates was used to monitor mitochondria, portions of the electron transport chain, and non-mitochondrial respiration as described in Supplementary Methods. O_2_ consumption was attributed to non-mitochondrial sources (“Non-Mito”), mitochondrial proton leak (“Proton Leak”), and basal or maximum mitochondrial respiratory rate (“Basal Rate” and “Max OCR”, respectively) as previously described [49,50].

Participants were also asked to complete a dietary recall questionnaire as part of the assessment of their nutritional status. The Block Brief Food Frequency Questionnaire (Block-FFQ) provided by NutritionQuest.com (Berkeley, CA, USA) was made available to participants online or in paper format midway through the study. Participants were instructed to answer the survey based on their typical consumption of food items. The data from this survey are presented in a truncated report focusing on micronutrient and macronutrient intake in Supplementary Methods and Supplementary Results.

### 2.4. Statistics

Statistical analyses were performed using SAS version 9.4 software (SAS Institute, Cary, NC). The study was designed to show a statistically significant (*p* < 0.025) 30% increase in vitamin/mineral concentrations with 80% power. To account for repeated measures within participants, a repeated measure in time design in PROC MIXED for blood variables was used. Fixed effects in the model were “treatment” (placebo or MV/MM), “timepoint” (initial/final), and their interaction, as well as the blocking variables “prior MV/MM use” (yes/no) and “BMI group” (normal/overweight/obese). The repeated measures within participants were modeled with an unrestricted variance-covariance matrix. To adjust the degrees of freedom for repeated measures, the Kenward–Rogers approximation for degrees of freedom was used.

The primary comparison was the change in the MV/MM group before and after treatment compared with the change in the placebo group before and after treatment. Tests for change in each group independently of the other were also performed. Effect modification by the blocking variables “prior MV/MM use” and “BMI” subgroup detected a statistically significant effect modification by prior MV/MM use; thus, MV/MM use by treatment was added to the final statistical model. In addition, effect modification by concurrent vitamin D use during the study detected a significant effect modification for vitamin D levels but no other vitamins and minerals; thus, current vitamin D use and the interaction between current vitamin D use and treatment were included in the final statistical model for vitamin D levels.

For the comparison of baseline values and comparison of the changes in both groups, PROC GLM was used with the aforementioned fixed effects. For comparison of changes in only one of the two treatment groups, PROC UNIVARIATE *t*-tests and Wilcoxon rank sum tests were used. All tests were two-sided. Significant group differences were determined if *p* ≤ 0.05. No adjustment was made for multiple comparisons.

## 3. Results

### 3.1. Healthy Older Men Have Suboptimal to Optimal Micronutrient Intake and Vitamin Biomarker Status

Initially, 63 older men met the screening criteria, and of those, 41 were enrolled in the study; 35 study participants completed the study (Figure 1). Since the enrollment criteria were designed to select a group of healthy older men, there were no issues of concern noted on the initial laboratory screens. MV/MM- and placebo-supplemented participants did not significantly differ in baseline characteristics and blood chemistry, except for higher plasma HDL cholesterol concentrations and lower systolic blood pressure and plasma triglyceride concentrations in the MV/MM compared with placebo-supplemented participants (Table 1). Despite these initial differences, no changes in laboratory screens were noted throughout the study in either group (Table S2).

Although diet composition was not a part of the enrollment criteria or the primary outcome of the study, a dietary assessment was performed during the study to survey eating patterns. Not surprisingly, many of the participants had intakes of several micronutrients that did not meet the recommended dietary allowance (RDA) for older adults (Tables S3 and S4), but no significant differences in micronutrient intake were noted between the MV/MM- and placebo-supplemented groups.

However, the use of dietary recall is notoriously problematic [51]. A better assessment of micronutrient status is through the use of blood micronutrient biomarkers. Therefore, the analysis focused on establishing the concentrations of several vitamins, nutritionally essential minerals, and carotenoids in blood samples (as described in Section 2.3 Methods). These blood concentrations were then assigned biomarker status categories: deficiency, insufficiency (for calcidiol only), and suboptimal status. While each of these categories is based on values established in the literature, it should be noted that the exact concentrations of “suboptimal” status are subject to debate. For this analysis, the definition of suboptimal status was blood micronutrient concentrations that were associated with an increased risk of mortality or morbidity but did not fall within the already pre-established deficiency (or insufficiency) range.

At enrollment, vitamin deficiencies in this cohort were exceptionally rare (Table 2). Two individuals assigned to the placebo group and three participants assigned to the MV/MM group were considered in deficiency at the start of the study (4 with phylloquinone and one with calcidiol). In addition, one participant in the placebo group and two in the MV/MM group had insufficient calcidiol levels. Interestingly, no participant showed multiple vitamin deficiencies and/or insufficiencies. By contrast, indicators of suboptimal vitamin status were common within the cohort (Table 2). At least half of the participants had suboptimal concentrations of ascorbic acid (57%), cobalamin (69%), and calcidiol (51%). Suboptimal blood concentrations of α-tocopherol (43%), pyridoxal phosphate (24%), phylloquinone (23%), and folate (20%) were also noted. However, no differences in biomarker status were observed between the placebo and MV/MM groups.

No participants from either group had blood concentrations of calcium, copper, magnesium, or zinc that would constitute a deficiency. Because suboptimal concentrations of these minerals are poorly defined, the assessment of suboptimal status was not performed.

### 3.2. MV/MM Supplementation Improves and/or Prevents Declines in Blood Vitamin Biomarker Status

**Figure 2:**
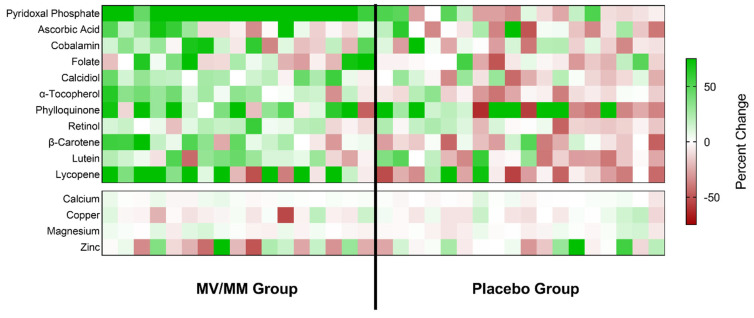
Changes in blood micronutrient concentrations for all study participants. The calculated percent change in blood concentrations of the indicated micronutrients is shown as a colored block for each participant in the study, with color saturation representing the degree of change during the study (green representing an increase over time; red representing a decline). The color saturation scale is capped at a change of ±75% so that changes for most micronutrients are visible.

To assess the impact of MV/MM intake, blood samples from study participants were analyzed after the supplementation period, and comparisons were made within and between groups (Table 3). To visualize changes within individuals that were assigned to the two study groups, the percent change in blood micronutrient concentrations is displayed as a heat map (Figure 2).

Overall, the greatest and most concerted change associated with MV/MM supplementation was an increase in plasma concentrations of pyridoxal phosphate. An increase in plasma concentrations of this vitamin was noted for all study participants in the MV/MM (range: +9.8 to +42.6 ng/mL), whereas no overall changes were observed in the placebo group (*p* = 0.62; Figure 2). MV/MM supplementation also improved blood biomarker concentrations of lipid-soluble vitamins and carotenoids in the majority of this group (Table 3; Figure 2). Specifically, 14 out of 17 individuals in the MV/MM group observed an increase in plasma α-tocopherol concentrations (*p* = 0.008). Additionally, plasma β-carotene concentrations increased in 13 out of 17 participants (*p* = 0.03), and 13 out of 17 individuals experienced increases in serum calcidiol (*p* = 0.0004). For calcidiol, specifically, it should be noted that these increased serum concentration levels came despite the concurrent use of vitamin D supplements.

By contrast, blood concentrations of these biomarkers declined in most men on the placebo supplement (Figure 2). Notably, plasma α-tocopherol concentrations decreased in 14 out of 18 placebo participants (*p* = 0.02). Similarly, 14 out of 18 placebo participants experienced a decline in plasma lycopene concentrations (*p* = 0.04).

MV/MM supplementation did not affect mineral blood biomarker concentrations in most participants (Table 3; Figure 2). Low vitamin status at baseline did modify some of the interactions observed in the treatment groups (see Supplementary Results). It should also be noted that a history of MV/MM supplement use showed significant interactions with plasma concentrations of retinol and magnesium. Those individuals who had no prior history of MV/MM use saw the most benefit from MV/MM supplementation (see Supplementary Results).

As expected, the changes in blood biomarker concentrations influenced the assessment of micronutrient status. Overall, most older men assigned to the MV/MM supplementation group during the study experienced an improvement in vitamin status (Table 4). Of those individuals taking the MV/MM and starting with non-optimal vitamin status (either suboptimal status, insufficiency, or deficiency), all but one (i.e., 15 of 16 participants) showed improvement in vitamin status of at least one vitamin by the end of the study (*p* = 0.0002). No participant in the MV/MM group showed blood vitamin concentrations indicative of deficiency or insufficiency at the end of the study. In the placebo group, only 7 of 17 participants starting with non-optimal vitamin status showed any improvement. Indeed, the number of individuals with vitamin insufficiency or deficiency only increased over time in the placebo group. Vitamin status declined in 13 of 18 placebo participants (compared with 2 of 17 MV/MM participants; *p* = 0.0005).

The changes in vitamin status did not seem to be specific to water-soluble or lipid-soluble vitamins, as similar changes were noted when status changes were separated into these categories. The general trends were towards increases in vitamin status for more individuals in the MV/MM group than the placebo group over time and that more individuals in the placebo group experienced a decline in vitamin status than in the MV/MM group by the end of the study (Supplementary Results). It was also more likely for individuals in the MV/MM group to experience status improvement in multiple vitamins, and it was more likely for individuals in the placebo group to experience a status decline in multiple vitamins over time.

### 3.3. MV/MM Supplementation Prevents Decline in Cellular O_2_ Consumption

**Figure 3:**
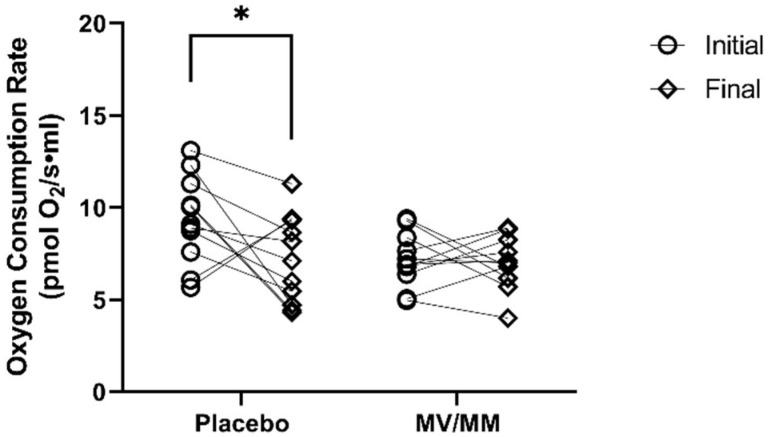
Oxygen consumption rate in isolated PBMCs. Total cellular oxygen consumption was measured before and after the supplementation period. Asterisks indicate significant changes in blood concentrations at initial to final time points.

In isolated PBMCs, there were no significant differences in monocyte respiratory capacity between treatment groups at the start of the study. After the supplementation period, however, cellular O_2_ consumption declined in 10 of 13 healthy older men on placebo (*p* = 0.02), a change that was not observed in the MV/MM supplementation group (*p* = 0.89; Figure 3). Similar changes were observed for the basal oxygen consumption rate (Table S5) but not for other markers of mitochondrial respiration. Thus, when compared to placebo, MV/MM supplementation increased cellular and basal rates of O_2_ consumption with no effect on other aspects of respiratory capacity, including mitochondrial proton leak and non-mitochondrial oxygen consumption.

## 4. Discussion

Older adults often take over-the-counter supplements to improve their health. However, previous studies on multivitamins show that they fall short of reducing chronic disease risk [6,23,28], leaving some vital questions as to the efficacy of an MV/MM supplement and its place in health promotion. Yet, despite decades of research, it is still not known if MV/MM supplements effectively change the nutrition status of older adults—a fundamental question that is needed to evaluate MV/MM studies in the future. Here, we present the results of a detailed study on MV/MM supplementation in a group of healthy older men, with the primary goal of assessing whether and how micronutrient status can be improved by MV/MM supplementation. The analysis shows that, despite the lack of overall micronutrient deficiencies in this population, MV/MM supplementation improves both blood concentrations and the status of many of these vitamins. In addition, the observation that PBMC O_2_ consumption is maintained by MV/MM supplementation, in contrast to placebo, may have important implications for maintaining mitochondrial function and energy metabolism with age.

These results appear to challenge the general perception of nutrition status in older adults. Older adults were selected for this trial because advanced age is generally considered to be a prescription for multiple vitamin and mineral deficiencies and would present an ideal cohort for an MV/MM intervention. This idea has been supported by previous reports on nutritional biomarkers, showing that overt micronutrient deficiencies are somewhat commonplace in adults over the age of 50 who do not regularly consume dietary supplements [5,8,24]. In the cohort presented here, however, despite every participant being above the age of 67 and the noted shortfalls in diet, no overarching blood micronutrient deficiencies were found. This is likely because the study design was biased towards a cohort of healthy older adults with few—if any—deficits of daily living and without overt disease diagnoses. Therefore, this is evidence to suggest that micronutrient deficiencies observed in prior reports may not be a function of age per se but a consequence of underlying health conditions or poor dietary habits. Previous reports showing the increased risk of vitamin deficiency in older adults, such as vitamin B_6_ [12,17], may include individuals with underlying health conditions, reduced food consumption, malabsorption syndromes, or other physiological limitations. However, given the size of this cohort, this will have to be explored in future studies.

Although deficiencies and insufficiencies were not common, suboptimal vitamin status, which is presently defined as having blood vitamin concentrations below optimum, was present in a significant number of healthy older adults. However, “optimal” status is a much more nuanced concept than deficiency since the health benefits of optimal versus suboptimal status are subtle and less precisely defined. There are some generally agreed-upon ideal blood concentrations for certain micronutrient biomarkers (such as vitamin C and vitamin D), but optimum blood concentrations for many vitamins are based on very limited scientific evidence. With these caveats in mind, 33 of 35 participants in this study were suboptimal in at least one of eight vitamins at baseline, despite consuming what appeared to be a rounded diet. Most common were shortfalls in three to five of eight vitamins (26 of 35 participants), with four having one suboptimal vitamin concentration and three having five suboptimal vitamin concentrations. This is where the impact of MV/MM supplementation is clear: those in the MV/MM group improved their vitamin status by the end of the study, whereas those in the placebo group either saw no change or had their vitamin status decline over time.

This study provides additional considerations for those designing MV/MM studies in the future. Unlike previous reports that focused on disease risk, this study did not examine disease-related outcomes. Instead, a focus was placed on a more fundamental question that relates MV/MM supplement use to improvement in micronutrient status. It is our position that this detailed approach is missing from many large MV/MM supplement trials. The results above show that the need for micronutrient supplementation varies by individual, and the response to the MV/MM intervention is equally variable. While some individuals in the MV/MM group improved their plasma vitamin concentrations dramatically upon supplementation, others only experienced marginal changes—thus, improvements to overall vitamin status were present but not universal. If consuming an MV/MM supplement does not guarantee changes in micronutrient status, how can one expect to observe changes in health outcomes? Based on these results, the recommendation would be to include micronutrient biomarkers as fundamental benchmarks in any clinical study on MV/MM supplements and to only look for changes in disease risk in those who showed improved micronutrient status.

Furthermore, instead of continuing with large and involved clinical trials that focus on MV/MM use and the reduction of chronic disease risk, it may be more prudent to focus on the ways MV/MM supplements influence markers of health status. Existing studies where relatively healthy older adults were the focus, such as the current study, have already provided evidence that daily MV/MM supplementation may improve indices of metabolism which potentially lowers the risk for pathophysiologies of aging. For example, a daily MV/MM supplement given to older adults limited plasma homocysteine levels, potentially through maintenance of the 1-carbon methyl cycle [52,53]. Additionally, a recently published 3-year trial showed that MV/MM supplements preserved global cognitive composite scores when compared to a placebo or a cocoa extract [54]. The data presented above shows that an MV/MM supplement may afford a benefit to metabolic maintenance, maintaining basal cellular O_2_ consumption and mitochondrial O_2_ respiratory characteristics in isolated PBMCs. Although follow-up studies that delineate which vitamin, mineral, or combination are involved in these effects are warranted, this provides a basis to determine the extent and precise nature of how MV/MM use influences health. Only after this groundwork is established can the role of MV/MM supplements as an intervention against chronic diseases of aging is elucidated.

Finally, this study had several strengths and weaknesses. This study was limited to participants within driving distance of the Oregon State University campus in Corvallis, Oregon. Thus, the study population was predominantly Caucasian (94%), all with post-high school education, and most (94%) with college degrees. They were also rigorously screened to exclude significant co-morbidities. Despite this, the dietary micronutrient intake and the prevalence of supplement use in these participants was similar to much larger trials with more diverse populations, as reported in NHANES data [23]. The use of a small population did allow a more in-depth examination of changes in micronutrient status after MV/MM supplementation with a focus on the individual. As subgroups that were analyzed (such as age subsets, BMI, and prior MV/MM use) comprised 10 individuals or less, these findings will need to be augmented by larger clinical trials. Another factor in this study is that while biomarker analysis is fundamental for understanding micronutrient supplementation studies, it also should be recognized that several minerals (cf. zinc) do not have adequate biomarkers. Finally, this study commenced just before and continued through the COVID-19 pandemic, which limited study participation and may have altered the lifestyle and diet of the remaining participants. No differences in subgroups that participated before COVID lockdowns versus those who participated afterward were noted, but caution is thus warranted in over-interpreting these results.

## 5. Conclusions

While healthy older adult men with an adequate diet show few actual vitamin and mineral deficiencies and inadequacies per se, the use of MV/MM supplements can improve or prevent declines in the status of several vitamins and may prevent declines in cellular bioenergetic status. Although MV/MM supplementation is a “one-size-fits-all” strategy and does not target specific micronutrient needs, it is a cost-effective approach to improve micronutrient status in older men and may have an as yet unappreciated impact on maintaining metabolic function in cells.

## Figures and Tables

**Table 1:** Baseline Characteristics of Participants Who Completed the Study.

Characteristic	Placebo (n = 18) ^1^	MV/MM (n = 17) ^1^	*p*-Value ^2^
Age, years	73 (5)	71 (3)	0.08
BMI, kg/m^2^	28.3 (3.6)	26.3 (3.6)	0.13
Systolic Blood Pressure, mm Hg	132 (14)	122 (10)	**0.03**
Diastolic Blood Pressure, mm Hg	77 (7)	75 (6)	0.32
Prior MV/MM use, n	8	5	0.49
Triglycerides, mg/dL ^3^	113 (52)	76 (30)	**0.02**
Total Cholesterol, mg/dL ^3^	179 (28)	178 (31)	0.93
HDL Cholesterol, mg/dL ^3^	53 (8)	66 (10)	**0.0002**
LDL Cholesterol, mg/dL ^3^	103 (26)	96 (25)	0.44
Glucose, mg/dL	105 (8)	100 (8)	0.10
Hb A1c, %	5.6 (0.3)	5.5 (0.3)	0.29
Ferritin, ng/mL	95 (73)	68 (31)	0.17

^1^ Values shown are means with standard deviations indicated in parentheses; ^2^ *p*-values are based on ANOVA for continuous data and Fisher’s Exact test for categorical data; *p*-values ≤ 0.05 are bolded for emphasis; ^3^ For these tests: Placebo (n = 18), MV/MM (n = 16).

**Table 2:** Micronutrient Status Categories in the Entire Cohort at Baseline.

Biomarker	Status Category ^1^	Participants ^2^
Retinol (Vitamin A)	Deficiency, <0.7 μM Suboptimal, <1.05 μM	0 (0%) 0 (0%)
Pyridoxal Phosphate (Vitamin B_6_)	Deficiency, <4.9 ng/mL Suboptimal, <8.9 ng/mL	0 (0%) 8 (24%)
Folate, RBC (Vitamin B_9_)	Deficiency, <150 ng/mL Suboptimal, <400 ng/mL	0 (0%) 7 (21%)
Cobalamin (Vitamin B_12_)	Deficiency, < 200 pg/mL Suboptimal, <500 pg/mL	0 (0%) 24 (69%)
Ascorbic Acid (Vitamin C)	Deficiency, <11.4 μM Suboptimal, <50 μM	0 (0%) 20 (57%)
Calicidiol (Vitamin D_3_)	Deficiency, <12 ng/mL Insufficiency, <20 ng/mL Suboptimal, <30 ng/mL	1 (3%) 3 (9%) 14 (40%)
α-tocopherol (Vitamin E)	Deficiency, <12 μM Suboptimal, <30 μM	0 (0%) 14 (43%)
Phylloquinone (Vitamin K)	Deficiency, <0.5 μM Suboptimal, <1.0 μM	4 (11%) 4 (11%)

^1^ Deficiency, insufficiency, and suboptimal vitamin status are based on published values; see methods for details. ^2^ Data represent the number of individuals in the cohort (n = 35), with percent shown in parentheses.

**Table 3:** Blood Micronutrient Concentrations at Baseline and Completion (Final) of the Supplementation Period for Participants Assigned Placebo and MV/MM.

Biomarker	Placebo Group (n = 18) ^1^		MV/MM Group (n = 17) ^1^		
	Baseline	Final	Baseline	Final	*p*-Value ^2^
Water-soluble Vitamins					
Pyridoxal Phosphate, ng/mL	14.6 (10.6)	15.2 (12.9)	11.9 (3.4)	28.7 (9.2)	<0.0001
Ascorbic Acid, μM	42 (14)	42 (19)	54 (18)	58 (14)	0.52
Cobalamin, pg/mL	485 (134)	501 (314)	428 (129)	480 (150)	0.39
Folate, RBC, ng/mL	537 (155)	504 (127)	640 (209)	695 (169)	0.16
Lipid-soluble Vitamins					
α-Tocopherol, μM	35 (9)	31 (6)	30 (9)	35 (9)	0.01
Calcidiol, ng/mL	28 (11)	27 (11)	32 (11)	38 (13)	0.0005
Phylloquinone, nM	2.0 (1.5)	1.9 (1.6)	1.5 (1.1)	2.0 (1.5)	0.80
Retinol, μM	6.2 (1.7)	5.8 (1.3)	6.3 (2.3)	6.7 (1.9)	0.06
Carotenoids					
β-Carotene, μM	0.41 (0.17)	0.36 (0.19)	0.39 (0.17)	0.45 (0.23)	0.01
Lutein, μM	1.8 (0.7)	1.7 (0.6)	1.9 (1.2)	2.0 (0.8)	0.18
Lycopene, μM	0.31 (0.23)	0.24 (0.14)	0.31 (0.29)	0.36 (0.29)	0.26
Minerals					
Calcium, μg/mL	55 (2)	55 (2)	55 (2)	56 (3)	0.13
Copper, μg/mL	0.62 (0.09)	0.62 (0.08)	0.66 (0.15)	0.61 (0.06)	0.41
Magnesium, μg/mL	11.5 (0.9)	11.4 (0.7)	11.6 (0.9)	11.7 (0.9)	0.47
Zinc, μg/mL	0.50 (0.12)	0.53 (0.13)	0.56 (0.13)	0.53 (0.16)	0.33

^1^ Values shown are means with standard deviations shown in parentheses. ^2^ *p*-values represent changes in micronutrient concentrations after ≥ 6 months of supplementation in the MV/MM group with adjustment for change in the placebo group, as described.

**Table 4:** Plasma Vitamin Status ^1^.

Biomarker	Status Category	Placebo (n = 18) ^2^		MV/MM (n = 17)	
		Initial	Final	Initial	Final
Retinol	Deficiency ^3^, <0.7 μM Suboptimal ^3^, <1.05 μM	0 (0%) 0 (0%)	0 (0%) 0 (0%)	0 (0%) 0 (0%)	0 (0%) 0 (0%)
Pyridoxal Phosphate	Deficiency, <4.9 ng/mL Suboptimal, <8.9 ng/mL	0 (0%) 5 (29%)	1 (6%) 6 (35%)	0 (0%) 3 (18%)	0 (0%) 0 (0%)
Folate, RBC	Deficiency, <150 ng/mL Suboptimal, <400 ng/mL	0 (0%) 5 (29%)	0 (0%) 5 (29%)	0 (0%) 2 (12%)	0 (0%) 0 (0%)
Cobalamin	Deficiency, <200 pg/mL Suboptimal, <500 pg/mL	0 (0%) 10 (56%)	0 (0%) 13 (73%)	0 (0%) 14 (82%)	0 (0%) 12 (71%)
Ascorbic Acid	Deficiency, <11.4 μM Suboptimal, <50 μM	0 (0%) 13 (73%)	1 (6%) 11 (61%)	0 (0%) 7 (41%)	0 (0%) 3 (18%)
Calcidiol	Deficiency, <12 ng/mL Insufficiency, <20 ng/mL Suboptimal, <30 ng/mL	1 (6%) 1 (6%) 9 (50%)	0 (0%) 6 (34%) 6 (34%)	0 (0%) 2 (12%) 5 (29%)	0 (0%) 0 (0%) 5 (29%)
α-tocopherol	Deficiency, <12 μM Suboptimal, <30 μM	0 (0%) 5 (28%)	0 (0%) 8 (44%)	0 (0%) 9 (53%)	0 (0%) 6 (35%)
Phylloquinone	Deficiency, <0.5 μM Suboptimal, <1.0 μM	1 (6%) 2 (11%)	0 (0%) 7 (39%)	3 (18%) 2 (12%)	0 (0%) 4 (24%)
	Total (at least one)				
	Deficiency	1 (6%)	2 (11%)	3 (18%)	0 (0%)
	Insufficiency	1 (6%)	3 (17%)	2 (12%)	0 (0%)
	Suboptimal	17 (94%)	13 (72%)	11 (65%)	15 (88%)

^1^ Data represents the number of individuals, with percent shown in parentheses. Treatment groups did not significantly differ in plasma vitamin status at baseline. The *p*-values for significant changes in vitamin status from placebo are as follows: retinol (*p* = 1), pyridoxal phosphate (*p* = 0.004), folate (*p* = 0.13), cobalamin (*p* = 0.12), ascorbic acid (*p* = 0.0492), calcidiol (*p* = 0.04), α-tocopherol (*p* = 0.19), and phylloquinone (*p* = 0.33). ^2^ For pyridoxal phosphate and RBC folate n = 17 for the placebo group and therefore n = 34 for both groups combined. ^3^ Deficiency, insufficiency, and suboptimal vitamin status are based on published values. See methods for details.

## Data Availability

The data are not publicly available due to privacy concerns. The data presented in this study are available on request from the corresponding author.

## Supplementary Materials

The following supporting information can be downloaded at: https://www.mdpi.com/article/10.3390/nu15122691/s1, Figure S1: MV/MM supplementation improves blood vitamin concentrations for individuals who had low concentrations at baseline; Table S1: Active Ingredients in Centrum Silver Men’s Formula; Table S2: Blood Chemistry Panel; Table S3: Self-Reported Macronutrient and Micronutrient Intake; Table S4: Estimated Daily Dietary Intake of Micronutrients by Study Participants; Table S5: Monocyte Respiratory Characteristics. (References [33,34,35,36,37,48,55] are cited in the Supplementary Materials).
