# Supplementary material for: Multivitamin/Multimineral Supplementation Prevents or Reverses Decline in Vitamin Biomarkers and Cellular Energy Metabolism in Healthy Older Men: A Randomized, Double-Blind, Placebo-Controlled Study
Source: Nutrients. 2023 Jun 9;15(12):2691. doi: 10.3390/nu15122691 (PMC10301451; doi:10.3390/nu15122691)
Supplement: Supplementary file 1 [file nutrients-15-02691-s001.zip › nutrients-2432439-supplementary.pdf]

Supplementary Methods

Study Design

This study is a single-center, randomized, double-blinded study designed to determine the impact of daily oral intake of an MV/MM supplement (Centrum Silver™ Men's Formula) by healthy, older adults for 6 months. While the primary outcome was to evaluate the impact of MV/MM supplementation on the participants' nutritional status, the secondary outcomes were to evaluate whether a change in nutritional status could impact metabolic energy production and/or cognitive function in older adult men. Since this manuscript is focused on one aspect of one of these secondary outcomes (metabolic energy production assessed by oxygen consumption rates), future publications may result from the analysis of this cohort.

Exclusion Criteria

Exclusion criteria were developed to avoid known micronutrient malabsorption issues, the presence of underlying disease, or conditions that might impact cognitive function. These are as follows:

- Currently taking supplements as recommended /ordered by a physician to correct a nutritional deficiency, **with an exception for vitamin D supplements**
- Current or past (two years) use of any tobacco products (including e-cigarettes or vaporizers)
- Dysphagia or difficulty swallowing.
- Bariatric (gastric bypass) surgery or serious chronic illness that might affect absorption of multivitamins or minerals such as Crohn's disease, celiac disease, chronic diarrhea, ulcerative colitis, gastritis, or a diagnosis of malabsorption syndrome.
- A diagnosis of cancer with chemotherapy or radiation treatment during the previous 5 years.
- Major surgery in the past 6 months or planned major surgery during the duration of the study.
- Prior heart attack, stroke, congestive heart failure, or chronic obstructive pulmonary disease.
- A current diagnosis of kidney, liver, or thyroid disease.
- Diabetes (Type 1 or Type 2)
- History of hypoglycemia or low blood sugar
- Current use of drugs to control blood sugar, thyroid medications, anti-seizure medication, anxiolytics, or antidepressants.
- BMI < 18.5 or ≥ 35 kg/m^2
- History of psychological or neurological disorders, or a diagnosis of dementia.
- Chronic migraines, i.e., more than fifteen headache days per month over a three month period of which more than eight are migraines
- Present treatment for drug or alcohol problems
- Habit of taking three or more alcoholic drinks per day
- Corrected visual acuity worse than 20/50
- Anyone who cannot handwrite a letter or move a computer joystick with one of their hands
- Cannot hear well enough to understand spoken instructions

Supplementation Protocol

Upon approval for entry into the study, the study statistician (Bobe, PhD) assigned participants to one of two intervention groups using a randomized block design. Participants were blocked by prior multivitamin use (yes, no) and BMI groups (<25 kg/m^2, 25-30 kg/m^2, >30 kg/m^2) into six homogeneous groups. Within their block, participants were assigned to one of two intervention groups using a random number generator in Microsoft Excel.

Bobe instructed the clinical nurse coordinator (Uesugi, RN) and clinical research coordinator (Michels, Ph.D.) to provide appropriately sealed bottles of tablets to participants. Only Bobe knew the identity of treatment assignments for the study but had no direct contact with study participants or the generation of data for analysis. The code linking participants with the intervention was opened after endpoint data for all participants were collected and analyzed. The total duration of study activities was approximately 30 months (November 2018 – May 2021), because the enrollment of participants was staggered as opposed to simultaneous, and also to account for accessibility problems related to the COVID-19 pandemic.

Study Visits, Adverse Effects Reporting, and Compliance

Before the supplementation started, participants gave fasting blood samples for measurement of baseline blood levels of micronutrients and an evaluation of oxygen consumption of isolated monocyte. Tablets were provided by Pfizer-GSK Consumer Healthcare (now Haleon PLC) in 90-count bottles that were sealed and coded without identifying marks on their contents. Participants were then advised to take one of the assigned tablets every day. The MV/MM contained all vitamins and nutritionally essential minerals (**Table S1**), except for iron and sodium, which are not recommended for older adults. In addition, the MV/MM contained lutein and lycopene, which are considered important for men's health.

Except for supplement use, participants were advised to continue their normal lifestyle during the study. This included no changes in diet and continuance of any habitual exercise or lack thereof. Participants were routinely asked throughout the study about noticeable effects or challenges to taking the supplements, and none were reported. Compliance was determined by pill counts – participants returned any partially-full or empty bottles at various points throughout the study. On average, compliance was excellent: those in the MV/MM group consumed 99% (±4%) of their tablets, while those in the placebo group consumed 95% (±7%) of tablets. Only three participants (all in the placebo group) were found to have compliance less than 90%.

Impact of the COVID-19 Pandemic

Shortly after recruiting and enrollment for the trial were concluded (March 2020), in-person clinical trial activities were suspended at Oregon State University due to the COVID-19 pandemic. Participants who were actively engaged in the study at that time were instructed to continue taking the supplements as directed until in-person activities could resume. If necessary, additional bottles of supplements were delivered to participants by study coordinators to allow compliance with the approved IRB protocol to continue. If an enrolled participant was not yet taking a supplement, they were instructed to maintain supplement restrictions until the time that these activities could resume.

In August 2020, a research resumption plan was approved by the IRB at Oregon State University to resume clinical trial activities. None of the modifications to the protocol altered the collection of samples for the primary and secondary outcomes, though some study participants declined participation due to the perceived risk of contracting COVID-19. However, during the clinical trial, no study participant or staff member reported any illness, nor did they test positive for SARS-CoV-2. Due to the delay in trial activities, the supplementation period in some individuals was longer than the 6-7 month target period. At the end of the study, participants in the MV/MM group took tablets for 239 ± 58 days while those in the placebo group took tablets for 234 ± 80 days. There were no significant differences noted in any measures reported here for individuals that completed the study before the pandemic versus those who completed it during the pandemic.

Blood Collection

Participants fasted overnight before blood collection. Blood was obtained at baseline and after intervention with the assigned supplement (at least 6 months of supplementation). Blood was processed immediately after collection and analyzed that day or stored at -80°C until analysis. Blood lipid panel (triglycerides, total cholesterol, HDL, VLDL, and LDL), comprehensive metabolic panel (CMP), Hb-A1C (a screening tool for participant inclusion), and ferritin were analyzed using standard clinical assays at the Student Health Services at Oregon State University (Corvallis, OR), a CLIA-certified diagnostic service, using ACE Axcel (spectrophotometric) and Tosoh 360AIA (Immunoenzymometric) analyzers. The results of this panel are provided in **Table S2**.

Biomarkers of Nutritional Status

Plasma concentrations of calcifediol (vitamin D), pyridoxal phosphate (B_6), cobalamin (B_{12}), and red blood cell folate (B_9) were determined by Quest Diagnostics (Seattle, WA), a CLIA-certified diagnostic lab. Other vitamins were measured at LPI's Analytical Services Core (a non-CLIA analytical facility where vitamins were quantified relative to standards).

Lipid-soluble Biomarkers: Plasma carotenoids (retinol, β-carotene, lycopene, lutein), phylloquinone, and α-tocopherol were analyzed using a Xevo MS/MS mass spectrometer (Waters Xevo TQD, Milford MA) housed in LPI's Analytical Services Core. Briefly, these analytes were extracted from plasma samples using hexane. The hexane extracts were dried to completion under the N_2 stream, reconstituted in methanol, and analytes chromatographically separated using a Waters Acquity BEH C-18 column (1.7 μm pore size, 2.1 x 100 mm column). Mass ions were analyzed by APCI UPLC-MS/MS in both positive and negative modes, and quantitated using authentic standards: α-tocopherol acetate, d_4-phylloquinone and carotenoids for vitamin E and carotenoids (1, 2), and vitamin K (3), respectively.

Ascorbic acid: Ascorbic acid was measured using the LPI's Analytical Services Core facilities using previously published methods (4). Briefly, blood plasma samples were acidified by mixing 1:1 with 10% (v/v) perchloric acid. The acidified extracts were injected into a Supelco LC-8 column (Millipore-Sigma, St Louis MO), and the analytes were separated by high-performance liquid chromatography. Sample peaks were detected using an electrochemical detector in the oxidizing mode at a potential of 500 mV. Ascorbic acid and uric acid were quantitated using authentic standards.

Minerals: Total plasma calcium, copper, zinc, iron, magnesium, and selenium were analyzed by inductively-coupled plasma-optical-emission spectroscopy (ICP-OES) in a non-CLIA-certified facility at OSU using a Spectros Arcos instrument (W.M. Keck Collaboratory for Plasma Spectrometry in the College of Earth, Ocean, and Atmospheric Sciences) as described in detail in other publications (5). Briefly, 100 μl of plasma were digested overnight in 1 ml nitric acid (OmniTrace® nitric acid, EMD Millipore, Billerica, MA) and diluted with Chelex-treated nanopure water to a final concentration of 10% v/v nitric acid followed by centrifugation to remove particulates. Each blood sample was analyzed in triplicate against known mineral standards. Because plasma selenium status fell below detection limits for most participants, it was not reported.

Mitochondrial Metabolic Assessment

For studies associated with mitochondrial metabolic assessment (a secondary outcome of the study), monocytes were isolated from 40 ml of heparinized blood at the time of collection. Peripheral blood monocytes were enriched from plasma using the ACCUSPIN System-Histopaque-1077 kit (Millipore-Sigma) following the manufacturer's instructions. Monocytes were kept on ice until use. All samples were obtained between 8:00 AM to 9:30 AM, and monocytes were used before noon on the same day.

High-resolution O_2 consumption measurements of mononuclear cells were conducted using the OROBOROS Oxygraph-2k and recorded by DatLab 4 software (OROBOROS Instruments, Innsbruck, Austria) using published methods (6). Isolated peripheral blood monocytes were resuspended in miR05 respiration buffer, then counted using a hemocytometer with Trypan blue (0.1%) for contrast. Following the determination of cell counts, 10^7 cells were added to the oxygraph cell chambers, and the volume was brought to 2 ml using miR05 buffer. Following equilibration to temperature (25^0C), cellular oxygen consumption was recorded. Monocytes were then permeabilized by the addition of digitonin (10 mg/ml stock) as shown by an initial loss of oxygen consumption to a basal rate. A sequential addition of the following substrates was provided (in final concentrations): ADP (2.5 mM), pyruvate + malate (10 and 4 mM, respectively), glutamate (10 mM), glycerol 3-phosphate (10 mM) to determine State 3 O_2 consumption contributed by TCA cycle, amino acid carbon skeletons, and the glycerol 3-phosphate shuttle, respectively. O_2 consumption linked to ATP production was determined by the addition of oligomycin (0.5 μM), which allowed for the determination of proton leak. FCCP was titrated (0.5 μM final concentration per addition) until maximal oxygen consumption was observed. Finally, antimycin A (2.5 μM) was added to the chambers to monitor O_2 consumption distinct from electron transport chain activity, reported as a non-mitochondrial O_2 consumption rate. Oxygen flux was expressed initially as pmol·sec^{-1}. Reported respiration rates were corrected for non-mitochondrial respiration by using the oxygen consumption rates determined after the addition of antimycin A.

Dietary Assessment

The study participants' dietary status was assessed using the Block Brief Food Frequency Questionnaire (Block-FFQ, NutritionQuest.com, Berkeley, CA). This survey was made available to participants online or on paper midway through the study. Participants were instructed to answer the survey based on their typical consumption of food items. Because the survey is provided online, participants could take it at home on their computers. Since the survey questions asked about the frequency of food consumption, the data reflects typical eating patterns. A truncated report of the Block-FFQ data is presented in **Table S3**.

The raw data from the Block FFQ showed some deficits in calorie consumption (range: 658 to 3560 kcal/day), which is common in dietary recall surveys. Thus, we adjusted the estimates of macronutrient and micronutrient intake relative to the estimated energy requirement (EER), using published equations (7) and the estimate of physical activity level provided by the Block FFQ. Although likely an overestimate of the total consumption of micronutrients by some individuals, the EER-adjusted values indicated the presence of dietary inadequacies. However, comparing the energy-adjusted micronutrient intakes from the Block FFQ with the Recommended Dietary Allowance (RDA) for older adults showed that most participants had caloric-adjusted intakes of lipid-soluble vitamins (vitamin D and E) and vitamin-like compounds (choline) below RDA (**Supplemental Methods; Table S4**). At least one-third of participants had caloric-adjusted intakes of minerals (potassium, calcium, and magnesium) below RDA.

Supplementary Results

Effects of Low Initial Vitamin Status

Three water-soluble vitamins and one lipid-soluble vitamin showed an interaction between baseline vitamin status and MV/MM supplementation (**Figure S1**). The overall trend for these interactions are that those individuals with the lowest blood concentrations of these vitamins at the start of the study showed improvements with MV/MM supplementation, but those with high initial concentrations did not. These are discussed in more detail below.

Ascorbic acid. Participants with suboptimal ascorbic acid (AA) status (defined as blood AA concentrations < 50 μM) increased their AA concentrations with MV/MM supplementation ($p=0.02$), whereas those with optimal AA status at enrollment did not ($p=0.33$; p interaction = 0.02; **Figure S1, Panel A**). MV/MM supplementation improved AA status (4 of 7 MV/MM participants with suboptimal levels improved and 0 of 17 declined in AA category) compared to placebo supplementation (2 of 12 placebo participants with suboptimal levels improved and 2 of 18 declined in AA category; $p = 0.049$).

Cobalamin. Participants with lower cobalamin concentrations (≤ 450 pg/mL) increased cobalamin concentrations after MV/MM supplementation ($p = 0.04$), whereas those with higher initial cobalamin values did not ($p = 0.53$; **Figure S1, Panel B**).

Folate. Participants with lower RBC folate concentrations (≤610 ng/mL) increased their folate concentrations with MV/MM supplementation ($p = 0.04$), whereas those with higher initial folate values did not ($p = 0.42$; **Figure S1, Panel C**).

Phylloquinone. Plasma phylloquinone concentration showed an interaction between baseline vitamin status and MV/MM supplementation. All 9 participants with lower phylloquinone concentrations (i.e. those below the median of 1.5 nmol/L) increased or kept their phylloquinone concentrations with MV/MM supplementation ($p = 0.008$), whereas those with higher initial folate values did not (4 of 8; $p = 0.57$; **Figure S1, Panel D**).

Effects of Prior MV/MM Use

In this cohort, 13 study participants (37%) reported using a daily MV/MM supplement during the initial screening. These individuals were instructed to terminate their supplement use (with an exception for vitamin D) at least 2 months before the start of the study to reduce the impact of MV/MM use. To equalize the effect of prior MV/MM use, these individuals were designated as a blocking group for randomization. While there were no concerted interaction effects between prior MV/MM use and treatment group assignment with respect to blood vitamin and mineral concentrations or changes in blood micronutrient concentrations over time, there are significant interactions noted for carotenoids and magnesium.

Vitamin A and carotenoids

Although there were no changes in plasma retinol concentrations overall, this seems to have been influenced by prior MV/MM use. In the placebo group, plasma retinol concentrations decreased only in those individuals who had previously used a daily MV/MM supplement ($p=0.03$). By contrast, participants in the MV/MM group who also had a history of MV/MM use maintained their retinol concentrations ($p=0.32$). Thus, after adjusting for changes in the placebo group, MV/MM treatment resulted in significantly higher plasma retinol concentrations in participants who had prior MV/MM use ($p=0.03$). No significant differences in plasma retinol concentrations were observed in participants without prior MV/MM use ($p=0.89$; treatment x MV/MM use interaction: $p=0.09$).

Our analysis also showed that prior MV/MM use may influence plasma lycopene and lutein concentrations, but the low concentrations normally present in the plasma and the variability in response to MV/MM treatment, combined with the influence of diet, make data interpretation difficult.

Magnesium/Minerals

Overall, blood mineral concentrations did not increase with MV/MM supplementation. However, there was a significant interaction between treatment and prior MV use for blood magnesium concentrations

(p=0.03). In the MV/MM group, blood magnesium concentration increased in only in participants who had no history of prior MV/MM use (p=0.04). No significant treatment differences were observed in participants who were assigned to the MV/MM group and also had a history of prior MV/MM use (p=0.26).

Vitamin Status by Vitamin Category

In the MV/MM group, the status of at least one water-soluble vitamin increased in 10 of 15 MV/MM participants with non-optimal vitamin status at baseline, while 10 of 13 individuals who had started the study with vitamin status below optimal showed an improvement after the supplementation period. Compared to 3 of 16 placebo participants who showed improvement in their status of water-soluble vitamins, this was a significant change (p = 0.01). Instead, the vitamin status of at least one water-soluble vitamin decreased in 8 of 18 placebo participants (compared with 1 of 17 MV/MM participants; p = 0.02).

To use pyridoxal phosphate as an illustrative example, PLP/vitamin B6 status increased in all MV/MM participants regardless of initial blood levels of this vitamin (Table 4; p = 0.004). To this end, all MV/MM participants were at optimal blood concentrations of pyridoxyl phosphate by the end of the study. In contrast, in the placebo group, of the 5 participants that had suboptimal levels of pyridoxal phosphate at the start of the study, 6 placebo-supplemented participants exhibited suboptimal values of vitamin B6 at the study's end, and 1 participant had now fallen into vitamin B6 deficiency.

For lipid-soluble vitamins, 10 of 13 individuals who had started the study with vitamin status below optimal showed an improvement after the supplementation period. However, of the 15 participants with non-optimal status of lipid-souble vitamins in the placebo group, only 4 of 15 individuals exhibited any improvement over time (p = 0.02). By contrast, the vitamin status of at least one fat-soluble vitamin decreased in 9 of 18 placebo participants (compared with 1 of 17 MV/MM participants; p = 0.007).

As another example, MV/MM supplementation also improved calcidiol status (4 of 7 MV/MM participants with suboptimal levels improved and 0 of 9 declined in the calcidiol category) compared to placebo supplementation (2 of 7 placebo participants with suboptimal levels improved and 6 of 11 declined the calcidiol category; p = 0.04).

References

1. Kopec, R. E., Schweiggert, R. M., Riedl, K. M., Carle, R., and Schwartz, S. J. (2013) Comparison of high-performance liquid chromatography/tandem mass spectrometry and high-performance liquid chromatography/photo-diode array detection for the quantitation of carotenoids, retinyl esters, alpha-tocopherol and phylloquinone in chylomicron-rich fractions of human plasma. *Rapid Commun Mass Spectrom* **27**, 1393-1402

2. Li, S., Tang, X., Lu, Y., Xu, J., Chen, J., and Chen, H. (2021) An improved method for the separation of carotenoids and carotenoid isomers by liquid chromatography-mass spectrometry. *J Sep Sci* **44**, 539-548

3. Usui, Y., Tanimura, H., Nishimura, N., Kobayashi, N., Okanoue, T., and Ozawa, K. (1990) Vitamin K concentrations in the plasma and liver of surgical patients. *Am J Clin Nutr* **51**, 846-852

4. Frei, B., England, L., and Ames, B. N. (1989) Ascorbate is an outstanding antioxidant in human blood plasma. *Proc Natl Acad Sci U S A* **86**, 6377-6381

5. Harrington, J. M., Young, D. J., Essader, A. S., Sumner, S. J., and Levine, K. E. (2014) Analysis of human serum and whole blood for mineral content by ICP-MS and ICP-OES: development of a mineralomics method. *Biol Trace Elem Res* **160**, 132-142

6. Jang, D. H., Shofer, F. S., Weiss, S. L., and Becker, L. B. (2016) Impairment of mitochondrial respiration following ex vivo cyanide exposure in peripheral blood mononuclear cells. *Clin Toxicol (Phila)* **54**, 303-307

7. Gerrior S., Juan W., and Basiotis, P. (2006) An easy approach to calculating estimated energy requirements. *Prev Chronic Dis* [serial online] Oct [*cited May 18, 2023*]. Available from: http://www.cdc.gov/pcd/issues/2006/oct/06_0034.htm

Table S1. Active Ingredients in Centrum Silver Men's Formula

Nutrient	Amount Per Tablet	% Daily Value	Nutrient	Amount Per Tablet	% Daily Value
Vitamin A	1050 mcg[1]	117%	Calcium	210 mg	16%
Vitamin C	120 mg	133%	Phosphorus	20 mg	2%
Vitamin D	25 mcg	125%	Iodine	150 mcg	100%
Vitamin E	27 mg	180%	Magnesium	75 mg	18%
Vitamin K	60 mcg	50%	Zinc	15 mg	100%
Thiamin	1.5 mg	125%	Selenium	21 mcg	38%
Riboflavin	1.7 mg	131%	Copper	0.5 mg	56%
Niacin	20 mg	125%	Manganese	4 mg	174%
Vitamin B_6	6 mg	353%	Chromium	60 mcg	171%
Folate	300 mcg	125%	Molybdenum	50 mcg	111%
Vitamin B_{12}	100 mcg	4167%	Chloride	72 mg	3%
Biotin	30 mcg	100%	Potassium	80 mg	2%
Pantothenic Acid	10 mg	200%	Nickel	5 mcg	-[2]
Lutein	300 mcg	-[2]	Silicon	2 mg	-[2]
Lycopene	600 mcg	-[2]	Vanadium	10 mcg	-[2]

[1] 29% of the Daily Value for vitamin A is present as β-carotene

[2] Daily Value not established

Table S2. Blood Chemistry Panel[1]

	Reference	Placebo Group Initial	Placebo Group Final	MV/MM Group Initial	MV/MM Group Final
Albumin	3.5-5.2 *g/dL*	4.3 (0.2) *g/dL*<br>*4-8 g/dL*	4.3 (0.2) *g/dL*<br>*3.9-4.6 g/dL*	*4.3 (0.2) g/dL*<br>*4-4.5 g/dL*	4.2 (0.3) *g/dL*<br>*3.8-4.8 g/dL*
Globulin	2.4-4.3 *g/dL*	2.7 (0.3) *g/dL*<br>*2.1-3.1 g/dL*	2.6 (0.4) *g/dL*<br>*2.1-3.0 g/dL*	2.6 (0.2) *g/dL*<br>*2.3-3.0 g/dL*	2.5 (0.2) *g/dL*<br>*2.2-2.9 g/dL*
Protein	6.4-8.3 *g/dL*	7.0 (0.3) *g/dL*<br>*6.3-7.4 g/dL*	6.9 (0.4) *g/dL*<br>*6.1-7.6 g/dL*	6.8 (0.2) *g/dL*<br>*6.5-7.3 g/dL*	6.7 (0.4) *g/dL*<br>*6.1-7.4 g/dL*
Alkaline Phosphatase	<147 *U/L*	60.7 (15) *U/L*<br>*35-88 U/L*	61.2(11.3) *U/L*<br>*44-79 U/L*	74.9 (21.8) *U/L*<br>*46-127 U/L*	72.8 (21.8) *U/L*<br>*43-140 U/L*
ALT (SGPT)	0-30 *U/L*	9.2 (7.0) *U/L*<br>*3-29 U/L*	8.9 (7.0) *U/L*<br>*3-30 U/L*	4.7 (3.0) *U/L*<br>*3-12 U/L*	9.4 (7.7) *U/L*<br>*3-30 U/L*
AST (SGOT)	0-31 *U/L*	14.4 (5.7) *U/L*<br>*8-34 U/L*	14.4 (5.9) *U/L*<br>*3-31 U/L*	13.9 (6.5) *U/L*<br>*3-26 U/L*	15.7 (7.1) *U/L*<br>*3-31 U/L*
Bilirubin	0.3-1.2 *mg/dL*	1.2 (0.4) *mg/dL*<br>*0.6-2.2 mg/dL*	1.2 (0.5) *mg/dL*<br>*0.7-2 mg/dL*	1.0 (0.4) *mg/dL*<br>*0.6-2.1 mg/dL*	1.0 (0.4) *mg/dL*<br>*0.7-1.7 mg/dL*
Total Cholesterol	<200 *mg/dL*	179 (28) *mg/dL*<br>*120-222 mg/dL*	174 (30) *mg/dL*<br>*140-245 mg/dL*	178 (31) *mg/dL*<br>*141-238 mg/dL*	181 (39) *mg/dL*<br>*127-242 mg/dL*
HDL Cholesterol	>40 *mg/dL*	53 (8) *mg/dL*<br>*39-68 mg/dL*	55 (11) *mg/dL*<br>*35-73 mg/dL*	66 (10) *mg/dL*<br>*50-80 mg/dL*	70 (13) *mg/dL*<br>*49-89 mg/dL*
LDL Cholesterol	<130 *mg/dL*	103 (26) *mg/dL*<br>*50-150 mg/dL*	99 (26) *mg/dL*<br>*71-165 mg/dL*	96 (25) *mg/dL*<br>*67-150 mg/dL*	97 (30) *mg/dL*<br>*61-161 mg/dL*
VLDL Cholesterol	<40 *mg/dL*	23 (11) *mg/dL*<br>*9-52 mg/dL*	21 (10) *mg/dL*<br>*11-46 mg/dL*	15 (6) *mg/dL*<br>*9-32 mg/dL*	15 (5) *mg/dL*<br>*9-30 mg/dL*
Triglycerides	<150 *mg/dL*	113 (52) *mg/dL*<br>*45-260 mg/dL*	107 (48) *mg/dL*<br>*54-232 mg/dL*	76 (30) *mg/dL*<br>*43-158 mg/dL*	71 (31) *mg/dL*<br>*8.3-151 mg/dL*
Creatinine	0.9-1.3 *mg/dL*	1.0 (0.2) *mg/dL*<br>*0.7-1.2 mg/dL*	1.1 (0.2) *mg/dL*<br>*0.8-1.5 mg/dL*	1.0 (0.1) *mg/dL*<br>*0.9-1.3 mg/dL*	1.1 (0.1) *mg/dL*<br>*0.9-1.4 mg/dL*
Glucose	70-99 *mg/dL*	105 (8) *mg/dL*<br>*89-120 mg/dL*	105 (11) *mg/dL*<br>*89-135 mg/dL*	100 (8) *mg/dL*<br>*84-118 mg/dL*	101 (5) *mg/dL*<br>*93-110 mg/dL*
BUN	6-20 *mg/dL*	16 (5) *mg/dL*<br>*8-29 mg/dL*	17 (4) *mg/dL*<br>*12-25 mg/dL*	16 (5) *mg/dL*<br>*8-26 mg/dL*	17 (5) *mg/dL*<br>*8-24 mg/dL*
Calcium	8.5-10.2 *mg/dL*	9.4 (0.3) *mg/dL*<br>*8.6-10 mg/dL*	9.5 (0.3) *mg/dL*<br>*8.9-10.2 mg/dL*	9.4 (0.3) *mg/dL*<br>*9.0-9.9 mg/dL*	9.4 (0.4) *mg/dL*<br>*8.8-10.1 mg/dL*
Sodium	136-145 *mmol/L*	140 (3) *mmol/L*<br>*133-144 mmol/L*	140 (3) *mmol/L*<br>*136-148 mmol/L*	141 (2) *mmol/L*<br>*138-144 mmol/L*	141 (3) *mmol/L*<br>*136-145 mmol/L*
Potassium	3.5-5.1 *mmol/L*	4.0 (0.5) *mmol/L*<br>*2.8-5 mmol/L*	4.2 (0.4) *mmol/L*<br>*3.5-5.3 mmol/L*	4.3 (0.3) *mmol/L*<br>*3.7-5.1 mmol/L*	4.5 (0.4) *mmol/L*<br>*3.9-5.3 mmol/L*
Chloride	98-107 *mmol/L*	103 (3) *mmol/L*<br>*96-110 mmol/L*	104 (4) *mmol/L*<br>*98-109 mmol/L*	105 (2) *mmol/L*<br>*101-109 mmol/L*	105 (2) *mmol/L*<br>*100-108 mmol/L*
Bicarbonate	22-31 *mmol/L*	27 (3) *mmol/L*<br>*20-32 mmol/L*	27 (3) *mmol/L*<br>*21-32 mmol/L*	27 (2) *mmol/L*<br>*25-30 mmol/L*	29 (2) *mmol/L*<br>*25-32 mmol/L*
Ferritin	22-340 *ng/mL*	95 (73) *ng/mL*<br>*13-236 ng/mL*	90 (78) *ng/mL*<br>*17-275 ng/mL*	68 (31) *ng/mL*<br>*23-132 ng/mL*	60 (27) *ng/mL*<br>*20-121 ng/mL*

[1] Data represent mean with standard deviation shown in parentheses and data range in italics. Biomarkers were determined by CLIA-certified laboratories as described in the Supplementary Methods. No significant differences were determined between groups during the study.

Table S3. Self-Reported Macronutrient and Micronutrient Intake[1]

	Unadjusted		EER[2]-Adjusted	
	Placebo (n=17)	MV/MM (n=16)	Placebo (n=17)	MV/MM (n=16)
Total Calories, *kcal*	1865 (414)	2052 (746)	2418 (316)	2496 (423)
	1180-2672	*658-3560*	*2009-3218*	*1935-3560*
EER, *kcal*	2381 (319)	2277 (289)	-	-
	2009-3218	*1935-2883*		
Total Fat, *g*	74 (22)	85 (38)	95 (15)	103 (27)
	41-116	*32-156*	*70-116*	*65-156*
Total Carbohydrates, *g*	220 (45)	232 (77)	288 (45)	286 (50)
	74-468	*76-379*	*218-444*	*213-379*
Protein, *g*	73 (19)	79 (38)	95 (24)	95 (29)
	35-107	*26-178*	*63-137*	*51-178*
Vitamin A, *mg RAE*	999 (514)	1012 (304)	1353 (790)	1265 (345)
	405-2536	*320-1384*	*597-3272*	*567-2117*
Thiamine, *mg*	1.6 (0.4)	1.6 (0.6)	2.1 (0.5)	1.9 (0.5)
	0.9-2.5	*0.5-3.0*	1.5-3.3	*1.4-3.0*
Riboflavin, *mg*	2.1 (0.5)	2.3 (0.9)	2.8 (0.8)	2.9 (0.7)
	1.3-3	*0.7-4.3*	*1.7-4.4*	*1.8-4.3*
Niacin, *mg NE*	21.0 (5.1)	21.7 (8.2)	27.8 (7.5)	26.3 (5.5)
	13.1-32.9	*5.0-41.5*	*16.4-47.7*	*15.3-41.5*
Pantothenic Acid, *mg*	5.4 (1.1)	6.2 (2.1)	7.2 (1.1)	7.7 (1.9)
	3.5-6.8	2.1-11.0	*4.7-11.8*	4.7-11.4
Vitamin B_6, *mg*	2.2 (0.6)	2.2 (0.7)	2.9 (0.6)	2.7 (0.7)
	1.3-3.5	*0.6-3.6*	*1.9-5.3*	*1.9-4.6*
Folate, *mcg*	572 (197)	553 (205)	761 (310)	695 (270)
	285-997	*152-953*	*381-1543*	*403-1535*
Vitamin B_{12}, *mcg*	6.0 (3.0)	5.6 (2.8)	8.0 (4.0)	6.7 (2.6)
	2.1-14.7	*1.3-10.7*	*2.2-17.2*	*2.2-10.7*
Vitamin C, *mg*	114 (41)	113 (52)	155 (71)	143 (53)
	54-220	*57-218*	*62-341*	*57-230*
Vitamin D, *mcg*	5.8 (2.9)	7.2 (4.4)	7.9 (4.4)	8.9 (5.1)
	0.8-12.3	*1.5-14.6*	*0.8-17.8*	*2.4-18.6*
Vitamin E, *mg*	8.8 (2.2)	10.5 (3.6)	11.6 (3.1)	13.0 (2.7)
	5.4-13.2	*4.6-17.5*	*7.9-20.6*	*8.3-17.6*
Vitamin K, *mcg*	191 (143)	243 (159)	262 (209)	300 (165)
	75-668	*84-688*	*79-865*	*133-688*
Choline, *mg*	317 (98)	354 (140)	416 (129)	433 (103)
	182-477	*145-669*	*246-663*	*251-669*
Calcium, *mg*	1097 (332)	1192 (563)	1459 (535)	1475 (527)
	394-1741	*449-2618*	*708-2743*	*699-2618*
Iron, *mg*	15.7 (6.1)	14.8 (5.9)	20.6 (8.2)	18.2 (5.0)
	7.1-31.4	*4.7-28.7*	*11.8-45.6*	*13.0-28.7*
Magnesium, *mg*	337 (97)	395 (113)	444 (123)	492 (69)

	181-634	*162-637*	*233-772*	*364-637*
Manganese, *mg*	4.1 (1.8)	4.7 (1.9)	5.3 (2.0)	5.9 (2.2)
	2.2-9.8	*1.6-8.6*	*2.6-9.9*	*2.5-11.2*
Phosphorus, *mg*	1384 (329)	1573 (596)	1820 (460)	1929 (448)
	636-1897	*527-2734*	*112-2871*	*1147-2734*
Potassium, *mg*	2983 (771)	3422 (959)	3964 (929)	4244 (600)
	1710-4479	*1203-5013*	*2781-5966*	*3485-5303*
Sodium, *mg*	3924 (949)	3394 (1403)	4269 (929)	4093 (937)
	1977-4978	*1276-6774*	*2781-5966*	*2712-6774*
Zinc, *mg*	11.4 (2.9)	12.7 (5.7)	14.9 (3.4)	15.4 (4.3)
	5.6-16.1	*4.2-28*	9.05-23.8	*10.6-28.0*
Copper, *mg*	1.5 (0.6)	1.6 (0.5)	1.9 (0.7)	2.0 (0.4)
	0.8-3.2	*0.8-2.4*	*1.1-3.7*	*1.4-2.7*
Selenium, *mcg*	95 (24)	100 (50)	125 (29)	120 (41)
	54-135	*31-241*	*88-184*	*52-241*

[1] Data represents the mean with standard deviation shown in parentheses and data range in italics.
[2] Estimated energy requirement (see Supplemental Methods).

Table S4. Estimated Daily Dietary Intake of Micronutrients by Study Participants

Micronutrient	Intake[1]	% below RDA[2]
Vitamin D, μ*g*	8.4 (4.7)	100% (33)
Vitamin E, *mg*	12.3 (3.0)	79% (26)
Choline, *mg*	424 (116)	85% (28)
Potassium, *mg*	4092 (941)	76% (25)
Calcium, *mg*	1464 (521)	33% (11)
Magnesium, *mg*	466 (102)	33% (11)
Vitamin A, *mg RAE*	1307 (607)	21% (7)
Vitamin C, *mg*	149 (62)	15% (5)
Pantothenic Acid, *mg*	7.4 (1.9)	12% (4)
Vitamin B_{12}, *mg*	7.4 (3.4)	12% (4)
Zinc, *mg*	15.1 (3.8)	9% (3)
Folate, *mg*	727 (288)	6% (2)
Selenium, μ*g*	122 (35)	3% (1)

[1] Values shown are means with standard deviations indicated in parentheses.
[2] Values shown are the percent of total participants with the number of individuals in parentheses; two individuals did not complete the nutrition survey.

Table S5. Monocyte Respiratory Characteristics

	Placebo (n=13)[1]		MV/MM (n=13)[1]		
	Initial	Final	Initial	Final	P-value[2]
Cellular	10.0 (3.9)	6.2 (3.3)	7.3 (3.6)	7.2 (3.4)	0.01
Basal Rate	13.0 (4.2)	9.5 (2.8)	10.4 (4.2)	10.5 (3.2)	0.02
Proton Leak	2.8 (2.0)	3.4 (2.8)	3.1 (2.1)	3.3 (1.5)	0.51
Non-mitochondrial	1.8 (1.0)	1.5 (0.6)	1.6 (0.6)	1.3 (0.6)	0.98
Maximum	34 (13)	27 (14)	29 (12)	28 (12)	0.38

[1] Data represents the mean oxygen consumption rate (pmol O_2/s•ml) for 10^6 cells. The standard deviation is shown in parentheses.

[2] p-values represent the change in the MV/MM group with adjustment for change in the placebo group.

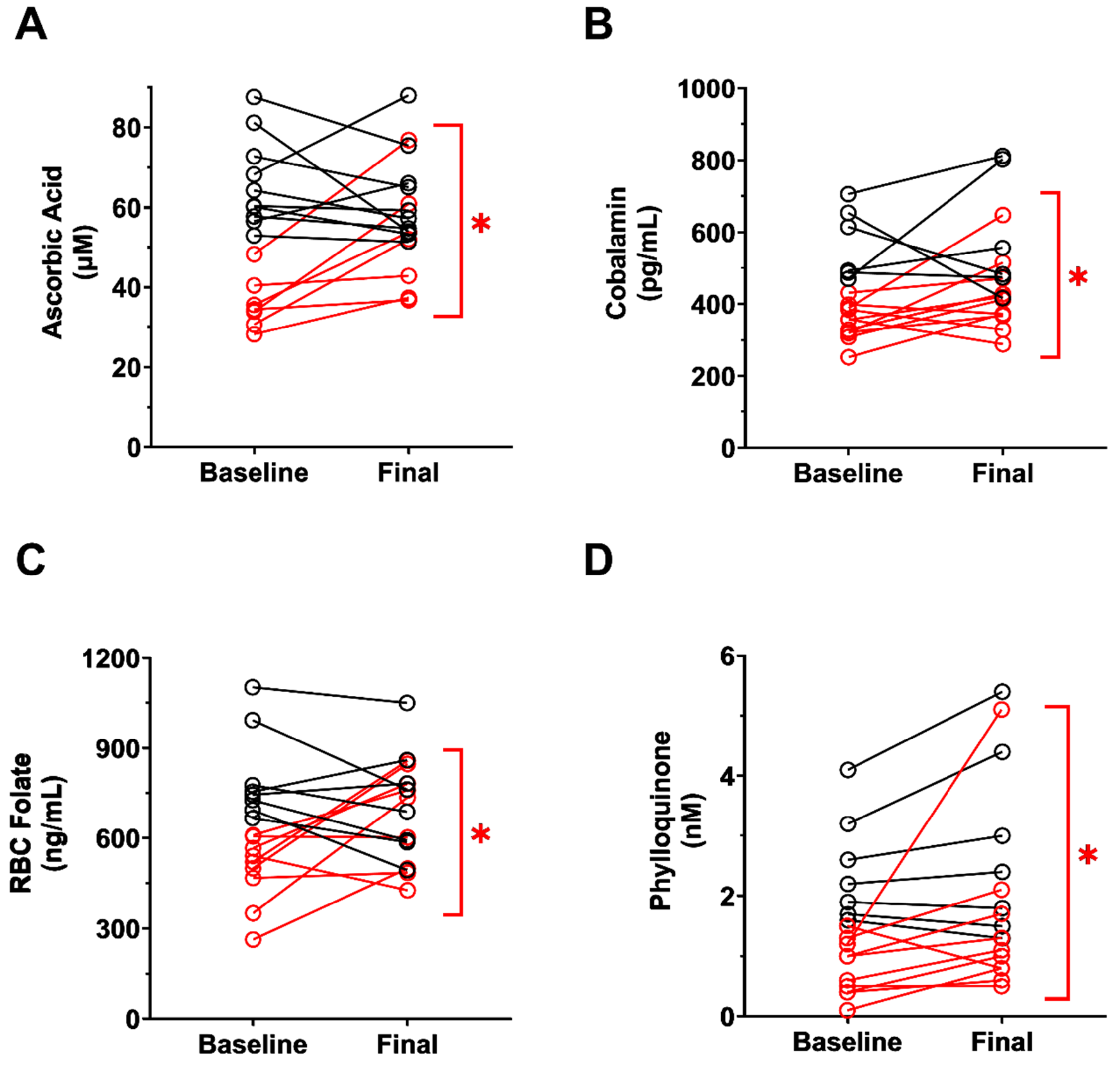

Figure S1. MV/MM supplementation improves blood vitamin concentrations for individuals who had low concentrations at baseline**.** Individuals who had A) suboptimal (<50 µM) plasma ascorbic acid status, B) suboptimal (<450 pg/mL) serum cobalamin status, C) the lower half of RBC folate concentrations (≤610 ng/mL), and D) the lower half of blood phylloquinone concentrations (1.5 ≤nM) at the initial assessment are shown in red. Asterisks indicate significant change from blood concentrations in final to initial time points, with p-values described in Supplemental Results.
